# Association between neighborhood socioeconomic status, built environment and SARS‐CoV‐2 infection among cancer patients treated at a Tertiary Cancer Center in New York City

**DOI:** 10.1002/cnr2.1714

**Published:** 2022-10-28

**Authors:** Shayan Dioun, Ling Chen, Grace Hillyer, Nicholas P. Tatonetti, Benjamin L. May, Alexander Melamed, Jason D. Wright

**Affiliations:** ^1^ Columbia Universtiy College of Physicians and Surgeons New York New York USA; ^2^ New York Presbyterian Hospital New York New York USA; ^3^ Mailman School of Public Health Columbia University New York New York USA; ^4^ Herbert Irving Comprehensive Cancer Center New York New York USA

**Keywords:** built environment, cancer, neighborhood socioeconomic status, SARS‐COV‐2

## Abstract

**Background:**

Racial and ethnic minority groups experience a disproportionate burden of SARS‐CoV‐2 illness and studies suggest that cancer patients are at a particular risk for severe SARS‐CoV‐2 infection.

**Aims:**

The objective of this study was examine the association between neighborhood characteristics and SARS‐CoV‐2 infection among patients with cancer.

**Methods and Results:**

We performed a cross‐sectional study of New York City residents receiving treatment for cancer at a tertiary cancer center. Patients were linked by their address to data from the US Census Bureau's American Community Survey and to real estate tax data from New York's Department of City Planning. Models were used to both to estimate odds ratios (ORs) per unit increase and to predict probabilities (and 95% CI) of SARS‐CoV2 infection. We identified 2350 New York City residents with cancer receiving treatment. Overall, 214 (9.1%) were infected with SARS‐CoV‐2. In adjusted models, the percentage of Hispanic/Latino population (aOR = 1.01; 95% CI, 1.005–1.02), unemployment rate (aOR = 1.10; 95% CI, 1.05–1.16), poverty rates (aOR = 1.02; 95% CI, 1.0002–1.03), rate of >1 person per room (aOR = 1.04; 95% CI, 1.01–1.07), average household size (aOR = 1.79; 95% CI, 1.23–2.59) and population density (aOR = 1.86; 95% CI, 1.27–2.72) were associated with SARS‐CoV‐2 infection.

**Conclusion:**

Among cancer patients in New York City receiving anti‐cancer therapy, SARS‐CoV‐2 infection was associated with neighborhood‐ and building‐level markers of larger household membership, household crowding, and low socioeconomic status.

**Novelty and impact:**

We performed a cross‐sectional analysis of residents of New York City receiving treatment for cancer in which we linked subjects to census and real estate date. This linkage is a novel way to examine the neighborhood characteristics that influence SARS‐COV‐2 infection. We found that among patients receiving anti‐cancer therapy, SARS‐CoV‐2 infection was associated with building and neighborhood‐level markers of household crowding, larger household membership, and low socioeconomic status. With ongoing surges of SARS‐CoV‐2 infections, these data may help in the development of interventions to decrease the morbidity and mortality associated with SARS‐CoV‐2 among cancer patients.

## 1 INTRODUCTION

Racial and ethnic minority groups experience a disproportionate burden of SARS‐CoV‐2 illness.^1^, ^2^ In New York City, the incidence of COVID‐19 varies substantially based on area of residence and is higher in neighborhoods traditionally characterized by lower socioeconomic status.^3^, ^4^ Additionally, there were two significant outbreaks (March–May and December) of COVID‐19 in 2020 in New York City.^5^ Previous studies suggest that cancer patients are at a particular risk for severe SARS‐CoV‐2 infection.^6^ Our objective was to determine the interplay of race and neighborhood socioeconomic factors on SARS‐CoV‐2 infection among oncology patients during a period of wide community spread of the virus.

## 2 METHODS

We performed a cross‐sectional study of residents of New York City receiving treatment for cancer at a tertiary cancer center from March 1, 2020 to December 31, 2020. Patients were identified by ICD‐10 cancer diagnosis codes in combination with billing codes for chemotherapy, targeted therapy or radiotherapy. SARS‐CoV‐2 status was determined by review of SARS‐CoV‐2 test results or documentation of infection from the medical record. Any SARS‐CoV‐2 test used to determine infection status was included in the study. Other factors extracted from the electronic medical record include age, sex, marital status, race/ethnicity, insurance status, cancer type, cancer treatment, and period when the patient tested positive for COVID (February–June, July–September, and October–December 2020). Subjects were linked by their primary address to the US Census Bureau's American Community Survey data, a nationwide survey including demographic, housing and socioeconomic data, and to real estate tax data from New York's Department of City Planning.^7^, ^8^ We abstracted each patient's building‐level characteristics, including assessed value (mean), residential units per building, and neighborhood level variables, including unemployment rate, racial and ethnic composition, median household income, percentage of families below the poverty rate, number of occupants per room (household crowding), average household size and membership, highest educational attainment, primary language spoken within the home, and population density of the neighborhood. New York City neighborhood tabulation areas were used to define a neighborhood.^8^

We fit unadjusted logistic regression models to estimate odds ratios (ORs) between each neighborhood socioeconomic and environment variable and SARS‐CoV‐2 infection, accounting for neighborhood clustering as described previously.^9^ We fit similar models further adjusting for patient characteristics selected on the basis of their a priori possibility of confounding the association. Analyses were conducted using SAS 9.4 (SAS Institute Inc., Cary, North Carolina). This study was approved by the Columbia University Institutional Review Board. The authors have no conflict of interest in relation to this work.

## 3 RESULTS

We identified 2489 New York City residents with cancer receiving treatment including 2350 (94.4%) that were linked to neighborhoods and buildings in the city. Overall, 214 (9.1%) were infected with SARS‐CoV‐2 (Table 1).

**TABLE 1:** Clinical and demographic characteristics of the overall cohort and COVID positive cohort

	Overall		COVID Positive	
	*N*	%	*N*	%
	2350	–	214	9.1
Age (Years)				
18–39	191	8.1	23	10.8
40–49	202	8.6	17	7.9
50–59	306	13.0	36	16.8
60–69	586	24.9	53	24.8
70–79	637	27.1	53	24.8
≥80	428	18.2	32	15.0
Sex				
Female	1294	55.1	109	50.9
Male	1056	44.9	105	49.1
Marital status				
Single	1319	56.1	121	56.5
Married	975	41.5	88	41.1
Other/unknown	56	2.4	5	2.3
Race				
White	715	30.4	39	18.2
Black	320	13.6	31	14.5
Hispanic	774	32.9	108	50.5
Asian, American Indian, Alaska, Pacific Islander	94	4.0	4	1.9
Other	139	5.9	12	5.6
Unknown	308	13.1	20	9.4
Insurance status				
None	9	0.4	1	0.5
Commercial	669	28.5	43	20.1
Medicare	1173	49.9	106	49.5
Medicaid	484	20.6	60	28.0
Other/unknown	15	0.6	4	1.9
Cancer type				
Head and neck	26	1.1	3	1.4
Gastrointestinal	317	13.5	41	19.2
Thoracic	205	8.7	11	5.1
Bone and soft tissue	13	0.6	1	0.5
Skin	56	2.4	5	2.3
Peripheral nerves and soft tissues	89	3.8	14	6.5
Breast	424	18.0	31	14.5
Gynecologic	167	7.1	14	6.5
Genitourinary	344	14.6	28	13
Brain and nervous system	102	4.3	6	2.8
Endocrine	27	1.2	1	0.5
Secondary cancers or unknown primary site	63	2.7	5	2.3
Hematologic	517	22.0	54	25.2
Treatment type				
Chemotherapy	2195	93.4	206	96.3
Radiation	483	20.6	50	23.4
Period when testing COVID positive (2020)				
February–June	–	–	145	67.8
July–September	–	–	25	11.7
October–December	–	–	44	20.6

**FIGURE 1:**
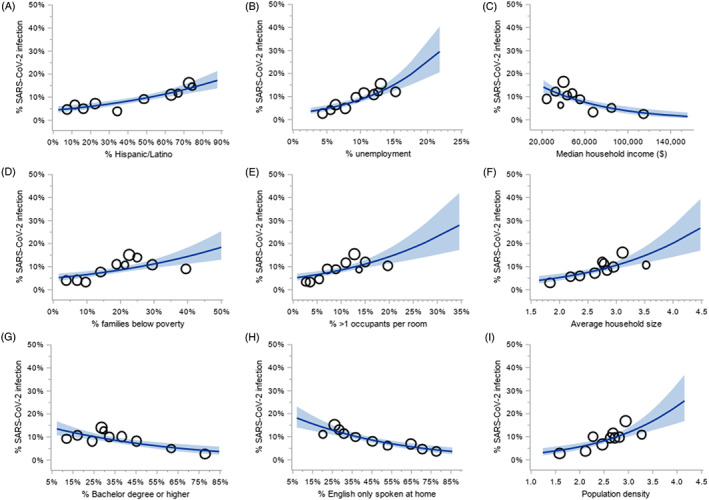
Built environment and neighborhood socioeconomic factors associated with SARS‐CoV‐2 infection among cancer patients getting treatment. The crude probability of detecting SARS‐CoV‐2 infection by neighborhood characteristics: (A) Percent of Hispanic/Latino population; (B) unemployment rate; (C) median household income; (D) percent of families below poverty line; (E) percent of greater than 1 occupant per room; (F) average household size; (G) percent of population with a bachelor degree or higher; (H) percent of population where English only spoken at home; (I) population density. Blue line indicated the predicted probability of SARS‐CoV‐2 infection from unadjusted logistic regression model. Shade indicated 95% confidence intervals. Bubbles indicated the mean of neighborhood characteristic by the observed SARS‐CoV‐2 infection rate of each decile, with bubble size proportionate to the number of patients in each decile

The likelihood of SARS‐CoV‐2 varied across measures of neighborhood socioeconomic status and built environment (Figure 1). The odds of SARS‐CoV‐2 infection were lowest in patients living in neighborhoods with higher median property values (OR per $100 000 increase = 0.88; 95% CI, 0.82–0.95), higher rates of bachelor degrees (OR per 1% increase = 0.98; 95% CI, 0.97–0.99), higher rates of English speaking only households (OR per 1% increase = 0.98; 95% CI, 0.97–0.99), higher rates of females compared to the total population (OR per 1% increase = 0.92; 95% CI, 0.86–0.99) and higher household income (OR = 0.98 per $1000 increase; 95% CI, 0.98–0.99). The odds of infection were higher in neighborhoods with higher Hispanic/Latino populations (OR per 1% increase = 1.02; 95% CI, 1.01–1.02), higher unemployment rates (OR per 1% increase = 1.13; 95% CI, 1.09–1.18), higher rates of poverty (OR per 1% increase = 1.03; 95% CI, 1.02–1.04), in neighborhoods with more households with >1 person per room (OR per 1% increase = 1.06; 95% CI, 1.03–1.09), increased average household size (OR = 2.13; 95% CI, 1.53–2.97) and higher population density (OR = 2.25; 95% CI, 1.60–3.17).

In adjusted models, the percentage of Hispanic/Latino population (aOR = 1.01; 95% CI, 1.005–1.02), unemployment rate (aOR = 1.10; 95% CI, 1.05–1.16), poverty rates (aOR = 1.02; 95% CI, 1.0002–1.03), rate of >1 person per room (aOR = 1.04; 95% CI, 1.01–1.07), average household size (aOR = 1.79; 95% CI, 1.23–2.59) and population density (aOR = 1.86; 95% CI, 1.27–2.72) were associated with SARS‐CoV‐2 infection (Supplemental Table).

## 4 DISCUSSION

Among cancer patients in New York City receiving anti‐cancer therapy, SARS‐CoV‐2 infection was associated with building‐ and neighborhood‐level markers of household crowding, larger household membership, and low socioeconomic status. With ongoing surges of SARS‐CoV‐2 infections, these data may help in the design and targeting of interventions to reduce the morbidity and mortality associated with SARS‐CoV‐2 among cancer patients.

Our study was limited to cancer patients in New York City and the findings may not be generalizable to other populations, there may be under capture of SARS‐CoV‐2 infection including asymptomatic infections, we lack data on the severity of infection, and there was significant correlation between neighborhood‐level variables which hinder multivariable analysis. However, this study suggests that differences in urban environment may be an important social determinant of SARS‐CoV‐2 transmission among cancer patients.

## AUTHOR CONTRIBUTIONS

**Shayan Dioun:** Conceptualization (equal); formal analysis (equal); investigation (equal); writing – original draft (equal); writing – review and editing (equal). **Ling Chen:** Data curation (equal); formal analysis (equal); methodology (equal); writing – original draft (equal); writing – review and editing (equal). **Grace Clarke Hillyer:** Conceptualization (equal); formal analysis (equal); investigation (equal); writing – review and editing (equal). **Nicholas Tatonetti:** Data curation (equal); formal analysis (equal); methodology (equal); writing – review and editing (equal). **Benjamin May:** Data curation (equal); formal analysis (equal); methodology (equal); writing – review and editing (equal). **Alexander Melamed:** Conceptualization (equal); formal analysis (equal); investigation (equal); writing – review and editing (equal). **Jason Wright:** Conceptualization (equal); formal analysis (equal); investigation (equal); supervision (equal); writing – original draft (equal); writing – review and editing (equal).

## CONFLICT OF INTEREST

The authors have stated explicitly that there are no conflicts of interest in connection with this article.

## ETHICS STATEMENT

Authors confirm that all procedures followed, were in accordance with the ethical standards with the Helsinki declaration of 1975, as revised in 2000.

## Supporting information


**Supplemental Table**. Multivariate logistic regression models for the association between built environment and neighborhood socioeconomic status and SARS‐CoV‐2 infection, adjusting for patient characteristics, and accounting for neighborhood clustering.

## Data Availability

The data that support the findings of this study are available on request from the corresponding author. The data are not publicly available due to privacy or ethical restrictions.
