# Supplementary material for: Association between neighborhood socioeconomic status, built environment and SARS‐CoV‐2 infection among cancer patients treated at a Tertiary Cancer Center in New York City
Source: Cancer Rep (Hoboken). 2022 Oct 28;6(2):e1714. doi: 10.1002/cnr2.1714 (PMC9874553; doi:10.1002/cnr2.1714)
Supplement: Supplementary file 1 — Supplemental Table. Multivariate logistic regression models for the association between built environment and neighborhood socioeconomic status and SARS‐CoV‐2 infection, adjusting for patient characteristics, and accounting for neighborhood clustering. [file CNR2-6-e1714-s001.docx]

Supplemental Table. Multivariate logistic regression models for the association between built environment and neighborhood socioeconomic status and SARS-CoV-2 infection, adjusting for patient characteristics, and accounting for neighborhood clustering.

|  | **aOR (95% CI)** | **aOR (95% CI)** | **aOR (95% CI)** | **aOR (95% CI)** | **aOR (95% CI)** | **aOR (95% CI)** | **aOR (95% CI)** | **aOR (95% CI)** | **aOR (95% CI)** |
| --- | --- | --- | --- | --- | --- | --- | --- | --- | --- |
| *Environment and neighborhood socioeconomic status* |  |  |  |  |  |  |  |  |  |
| % Hispanic/Latino, increase of 1% | 1.01 (1.005-1.02) | - | - | - | - | - | - | - | - |
| % unemployment, increase of 1% | - | 1.10 (1.05-1.16) | - | - | - | - | - | - | - |
| Median household income, increase of 1000$ | - | - | 0.99 (0.98-0.996) | - | - | - | - | - | - |
| % families below poverty, increase of 1% | - | - | - | 1.02 (1.0002-1.03) | - | - | - | - | - |
| % >1 occupants per room, increase of 1% | - | - | - | - | 1.04 (1.01-1.07) | - | - | - | - |
| Average household size, increase of 1 | - | - | - | - | - | 1.79 (1.23-2.59) | - | - | - |
| % Bachelor degree or higher, increase of 1% | - | - | - | - | - | - | 0.99 (0.98-0.999) | - | - |
| % English only spoken at home, increase of 1% | - | - | - | - | - | - | - | 0.98 (0.97-0.99) | - |
| Population density, increase of 1 | - | - | - | - | - | - | - | - | 1.86 (1.27-2.72) |
| *Age, increase of 1 year* | 0.99 (0.98-1.002) | 0.99 (0.98-1.003) | 0.99 (0.98-1.003) | 0.99 (0.98-1.003) | 0.99 (0.98-1.003) | 0.99 (0.98-1.003) | 0.99 (0.98-1.003) | 0.99 (0.98-1.002) | 0.99 (0.98-1.003) |
| *Sex* |  |  |  |  |  |  |  |  |  |
| Female | Referent | Referent | Referent | Referent | Referent | Referent | Referent | Referent | Referent |
| Male | 1.13 (0.80-1.62) | 1.16 (0.82-1.66) | 1.15 (0.81-1.64) | 1.15 (0.81-1.64) | 1.14 (0.80-1.63) | 1.14 (0.80-1.63) | 1.14 (0.80-1.63) | 1.14 (0.80-1.62) | 1.15 (0.81-1.64) |
| *Marital status* |  |  |  |  |  |  |  |  |  |
| Single | Referent | Referent | Referent | Referent | Referent | Referent | Referent | Referent | Referent |
| Married | 1.22 (0.90-1.67) | 1.22 (0.89-1.67) | 1.20 (0.88-1.63) | 1.20 (0.88-1.64) | 1.18 (0.87-1.61) | 1.16 (0.85-1.59) | 1.18 (0.87-1.61) | 1.19 (0.88-1.63) | 1.16 (0.85-1.58) |
| Other/unknown | 1.23 (0.46-3.27) | 1.29 (0.48-3.42) | 1.20 (0.45-3.20) | 1.20 (0.45-3.19) | 1.19 (0.45-3.15) | 1.16 (0.44-3.07) | 1.18 (0.45-3.14) | 1.21 (0.46-3.21) | 1.17 (0.44-3.10) |
| *Race* |  |  |  |  |  |  |  |  |  |
| White | Referent | Referent | Referent | Referent | Referent | Referent | Referent | Referent | Referent |
| Black | 1.56 (0.92-2.62) | 1.30 (0.76-2.23) | 1.36 (0.79-2.34) | 1.54 (0.90-2.63) | 1.65 (0.98-2.77) | 1.60 (0.95-2.69) | 1.48 (0.87-2.54) | 1.78 (1.06-2.99) | 1.59 (0.94-2.67) |
| Hispanic | 1.84 (1.17-2.90) | 1.83 (1.17-2.87) | 1.97 (1.27-3.06) | 2.22 (1.44-3.43) | 2.23 (1.46-3.40) | 2.23 (1.47-3.39) | 2.16 (1.40-3.34) | 2.01 (1.31-3.08) | 2.18 (1.43-3.32) |
| Asian, American Indian, Alaska, Pacific island | 0.72 (0.24-2.09) | 0.75 (0.26-2.21) | 0.68 (0.23-2.00) | 0.72 (0.25-2.11) | 0.67 (0.23-1.98) | 0.69 (0.24-2.03) | 0.69 (0.23-2.01) | 0.58 (0.20-1.71) | 0.70 (0.24-2.05) |
| Other | 1.24 (0.61-2.53) | 1.19 (0.59-2.42) | 1.22 (0.60-2.48) | 1.34 (0.66-2.72) | 1.33 (0.66-2.68) | 1.31 (0.65-2.63) | 1.27 (0.63-2.58) | 1.26 (0.62-2.55) | 1.30 (0.64-2.61) |
| Unknown | 0.95 (0.53-1.71) | 0.88 (0.49-1.59) | 0.93 (0.52-1.68) | 1.01 (0.56-1.82) | 1.04 (0.58-1.85) | 1.04 (0.59-1.85) | 0.99 (0.55-1.79) | 1.00 (0.56-1.77) | 1.03 (0.58-1.83) |
| *Insurance status* |  |  |  |  |  |  |  |  |  |
| Commercial | Referent | Referent | Referent | Referent | Referent | Referent | Referent | Referent | Referent |
| Medicare | 1.44 (0.93-2.22) | 1.41 (0.91-2.19) | 1.39 (0.90-2.15) | 1.42 (0.92-2.20) | 1.43 (0.92-2.22) | 1.43 (0.93-2.22) | 1.43 (0.92-2.21) | 1.42 (0.92-2.20) | 1.43 (0.92-2.21) |
| Medicaid | 1.47 (0.95-2.28) | 1.48 (0.95-2.28) | 1.43 (0.93-2.22) | 1.47 (0.95-2.28) | 1.46 (0.95-2.27) | 1.48 (0.96-2.28) | 1.48 (0.96-2.29) | 1.44 (0.93-2.23) | 1.47 (0.95-2.28) |
| None | 1.72 (0.19-15.58) | 1.78 (0.20-16.15) | 1.79 (0.20-16.27) | 1.75 (0.19-15.93) | 1.79 (0.20-16.25) | 1.81 (0.20-16.51) | 1.87 (0.20-17.04) | 1.73 (0.19-15.74) | 1.84 (0.20-16.81) |
| Other/unknown | -ⱡ | -ⱡ | -ⱡ | -ⱡ | -ⱡ | -ⱡ | -ⱡ | -ⱡ | -ⱡ |
| *Cancer site* |  |  |  |  |  |  |  |  |  |
| Head and neck | 1.61 (0.43-6.06) | 1.59 (0.42-5.99) | 1.60 (0.42-6.07) | 1.66 (0.44-6.27) | 1.69 (0.44-6.39) | 1.70 (0.45-6.42) | 1.62 (0.43-6.16) | 1.67 (0.44-6.30) | 1.67 (0.44-6.32) |
| Gastrointestinal | 1.73 (0.99-3.03) | 1.71 (0.97-3.00) | 1.72 (0.98-3.02) | 1.76 (1.003-3.09) | 1.80 (1.03-3.17) | 1.76 (1.01-3.09) | 1.73 (0.99-3.04) | 1.75 (1.0003-3.07) | 1.77 (1.01-3.11) |
| Thoracic | 0.71 (0.33-1.51) | 0.70 (0.33-1.50) | 0.70 (0.33-1.50) | 0.71 (0.33-1.52) | 0.72 (0.34-1.53) | 0.72 (0.34-1.53) | 0.70 (0.33-1.49) | 0.71 (0.34-1.52) | 0.72 (0.34-1.53) |
| Bones and soft tissues | 1.16 (0.14-9.80) | 1.10 (0.13-9.19) | 0.99 (0.12-8.33) | 1.03 (0.12-8.67) | 1.00 (0.12-8.42) | 0.90 (0.11-7.55) | 0.97 (0.12-8.18) | 0.94 (0.11-8.02) | 0.91 (0.11-7.62) |
| Skin | 0.92 (0.20-4.17) | 0.92 (0.20-4.15) | 0.95 (0.21-4.31) | 0.95 (0.21-4.31) | 0.95 (0.21-4.32) | 0.97 (0.21-4.37) | 0.94 (0.21-4.26) | 0.95 (0.21-4.31) | 0.97 (0.21-4.39) |
| Peripheral nerves and soft tissues | 2.66 (1.29-5.51) | 2.59 (1.25-5.37) | 2.56 (1.23-5.29) | 2.55 (1.23-5.28) | 2.57 (1.24-5.31) | 2.49 (1.21-5.15) | 2.52 (1.22-5.22) | 2.60 (1.26-5.38) | 2.51 (1.21-5.18) |
| Breast | Referent | Referent | Referent | Referent | Referent | Referent | Referent | Referent | Referent |
| Gynecologic | 1.24 (0.63-2.45) | 1.22 (0.62-2.42) | 1.27 (0.65-2.51) | 1.29 (0.65-2.54) | 1.30 (0.66-2.55) | 1.28 (0.65-2.53) | 1.27 (0.65-2.51) | 1.27 (0.65-2.51) | 1.27 (0.65-2.51) |
| Male genital organs | 0.96 (0.46-2.00) | 0.95 (0.45-2.00) | 0.97 (0.46-2.04) | 0.97 (0.46-2.04) | 0.99 (0.47-2.09) | 1.00 (0.47-2.09) | 0.99 (0.47-2.07) | 0.98 (0.47-2.05) | 0.99 (0.47-2.07) |
| Urinary tract | 1.61 (0.73-3.57) | 1.58 (0.71-3.50) | 1.55 (0.70-3.45) | 1.53 (0.69-3.40) | 1.57 (0.71-3.47) | 1.57 (0.71-3.49) | 1.56 (0.70-3.46) | 1.60 (0.72-3.55) | 1.59 (0.71-3.52) |
| Brain and nervous system | 0.87 (0.34-2.25) | 0.86 (0.33-2.21) | 0.85 (0.33-2.19) | 0.85 (0.33-2.19) | 0.87 (0.34-2.24) | 0.86 (0.34-2.22) | 0.86 (0.33-2.21) | 0.86 (0.33-2.21) | 0.86 (0.33-2.21) |
| Endocrine | 0.47 (0.06-3.79) | 0.49 (0.06-4.01) | 0.47 (0.06-3.86) | 0.48 (0.06-3.89) | 0.50 (0.06-4.07) | 0.49 (0.06-4.01) | 0.47 (0.06-3.90) | 0.47 (0.06-3.77) | 0.49 (0.06-3.97) |
| Kaposi Sarcoma | -ⱡ | -ⱡ | -ⱡ | -ⱡ | -ⱡ | -ⱡ | -ⱡ | -ⱡ | -ⱡ |
| Secondary cancers or unknown primary | 1.34 (0.48-3.74) | 1.37 (0.49-3.84) | 1.28 (0.46-3.59) | 1.29 (0.46-3.60) | 1.26 (0.45-3.52) | 1.24 (0.44-3.45) | 1.28 (0.46-3.58) | 1.28 (0.46-3.57) | 1.24 (0.44-3.46) |
| Hematologic | 1.52 (0.90-2.57) | 1.57 (0.93-2.65) | 1.56 (0.92-2.64) | 1.57 (0.93-2.66) | 1.59 (0.94-2.69) | 1.60 (0.95-2.71) | 1.57 (0.93-2.66) | 1.54 (0.92-2.61) | 1.60 (0.95-2.71) |
| *Chemotherapy* |  |  |  |  |  |  |  |  |  |
| No | Referent | Referent | Referent | Referent | Referent | Referent | Referent | Referent | Referent |
| Yes | 2.46 (1.10-5.51) | 2.45 (1.09-5.49) | 2.39 (1.07-5.35) | 2.41 (1.08-5.40) | 2.39 (1.07-5.34) | 2.40 (1.07-5.36) | 2.41 (1.08-5.39) | 2.44 (1.09-5.47) | 2.39 (1.07-5.35) |
| *Radiation* |  |  |  |  |  |  |  |  |  |
| No | Referent | Referent | Referent | Referent | Referent | Referent | Referent | Referent | Referent |
| Yes | 1.48 (1.004-2.19) | 1.47 (0.99-2.18) | 1.45 (0.98-2.15) | 1.48 (0.998-2.18) | 1.46 (0.99-2.17) | 1.50 (1.01-2.21) | 1.48 (0.9997-2.19) | 1.48 (1.003-2.19) | 1.50 (1.01-2.21) |

ⱡ Unestimable
